# Feasibility of a physical activity intervention for children and adolescents with anxiety and depression

**DOI:** 10.1186/s40814-024-01466-8

**Published:** 2024-03-05

**Authors:** Arne Kodal, Fiona Muirhead, John J. Reilly, Gro Janne Wergeland, Paul Joachim Bloch Thorsen, Lars Peder Bovim, Irene Bircow Elgen

**Affiliations:** 1 Department of Child and Adolescent Psychiatry, Division of Psychiatry, Haukeland University Hospital, N-5021 Bergen, Norway; 2 Norce Research, RKBU Vest-Regional Centre for Child and Youth Mental Health and Child Welfare, Postboks 22, Nygårdstangen, Bergen, 5838 Norway; 3 School of Psychological Science and Health, University of Strathclyde, Glasgow, Scotland; 4 Department of Clinical Medicine, Faculty of Medicine, University of Bergen, N-5020 Bergen, Norway; 5 Department of Health and Functioning, Western Norway University of Applied Sciences, Bergen, Norway

**Keywords:** Anxiety, Depression, Youth, Physical activity, Intervention

## Abstract

**Background:**

Physical activity is identified as a key modifiable factor towards good short- and long-term mental health and has shown positive effects on anxiety and depression in children and adolescents. However, physical activity-based interventions are not a part of standard mental health care and evidence on the effect of such interventions is still lacking. A transdiagnostic, physical activity-based intervention was developed as a supplement to routine clinical care for youth in specialized child and adolescent mental health services.

**Methods:**

/design.

The feasibility of the physical activity intervention (Confident, Active, and Happy Youth) was evaluated in an open-label study by assessing the recruitment process, acceptability, intervention suitability, contentment, and preliminary intervention effects in the form of youth and parent-rated anxiety and depressive symptoms. Physical activity levels were objectively measured using Actigraph™ physical activity sensors, and progression to a definitive study was evaluated in accordance with a priori criteria.

**Results:**

In total 21 of 25 eligible youth consented to participate, two dropped out of the intervention and 19 completed (76% of eligible participants). The retention rate among consenting participants was 89% and mean attendance to sessions was 83%. The suitability of the intervention was rated as good by the youth and their parents, and intervention contentment was rated high. Changes in youth and parent-rated symptom measures following the intervention were negligible, except for parent-rated anxiety symptoms assessed at 10-month follow-up. Accelerometer data indicated lower levels of moderate to vigorous activity during sessions than intended. No adverse effects were noted.

**Conclusion:**

This feasibility study met the pre-determined progression criteria to a definitive study. Thus, a larger trial with longer follow-up should be conducted to explore the effect of the intervention.

**Trial registration:**

ClnicalTrials.gov, NCT05049759. Retrospectively registered, 20.09.2021.

**Supplementary Information:**

The online version contains supplementary material available at 10.1186/s40814-024-01466-8.

## Key messages regarding feasibility

- What uncertainties existed regarding the feasibility? It was unclear if we could recruit participants if they would participate in and complete the intervention.
- What are the key feasibility findings? We were able to recruit participants, treatment was assessed as acceptable, suitable, and practical, and participants and parents reported high degrees of contentment with the intervention. Procedural issues hindered the collection of activity data.
- What are the implications of the feasibility findings for the design of the main study? While all pre-determined progression criteria were met, findings indicate the need for some adjustment to measures and the need for a broad multi-faceted assessment of outcome.

## Background

Anxiety and depression commonly develop early in life, and short- and long-term consequences include reduced quality of life, psychiatric and somatic comorbidity, disability, loss of education and/or work, suicide, and reduced lifespan [1]. Rates of anxiety and depression in children and adolescents are increasing [2], and even when provided with the best available treatment for these disorders, post-treatment remission rates remain just slightly above chance, e.g., 50% [3]. Such suboptimal treatment outcomes have large consequences for the individual, their families, and society and place a large strain on treatment services. As such, there is a pressing need for the development of new and supplementary interventions to improve recovery rates, prevent relapse and development of future comorbidity, and help mitigate the current load on mental health services [3, 4].

Physical activity (PA) has been indicated as a potent modifiable factor towards effective short- and long-term mental health [5, 6]. Evidence is scarce but accumulating. A recent meta-analysis examining the effect of PA on anxiety in young people (mean age 14.2–25 years) found a moderate improvement in state anxiety (SMD =  − 0.55, 95% CI − 0.77, − 0.32, *p* < 0.01) compared to control groups [7]. Regarding depression and depressive symptoms, meta-analyses and a systematic review of meta-analyses have identified a small to moderate effect of PA on depressive symptoms [8–12].

However, despite these encouraging findings, very few data exist regarding the effect of PA as a supplementary treatment targeting multiple psychiatric disorders in clinical populations, e.g., anxiety and depression. Psychiatric comorbidity is the rule, rather than the exception [13] and nearly 75% of youth with depression have a comorbid anxiety disorder [14]. Anxiety and depression share a number of core symptoms, including reduced or low levels of physical activity; lack of confidence in one’s ability to cope with situations that incite distress and/or fear; decreased willingness to engage in and avoidance of situations that may incite distress and/or fear and lowered mood [15, 16]. Thus, addressing these disorders co-jointly broadens the possible applicability of an intervention, while also reflecting current trends of comorbidity in youth populations.

In sum, physical activity-based interventions are a viable yet under-researched and under-utilized approach that may address the rising rates of mental health problems in youth and supplement existing treatment. Against this backdrop, we have developed a supplementary transdiagnostic, physical activity-based intervention for youth with anxiety and depression receiving treatment at specialized child and adolescent mental health services. The intervention is called Confident, Active, and Happy Youth (CAHY) and is described in a previous article (Kodal et al. 2022). In the present study, we test the feasibility of the CAHY intervention, in line with The British *Medical Research Council* guidance (MRC: Skivington et al. 2021). The MRC framework has four stages: development, feasibility/piloting, evaluation and implementation/upscaling, and the framework strongly advises carrying out feasibility and pilot work prior to running a definitive trial [17]. The focus of the present study will be on identifying uncertainties that may negatively influence a future definitive trial. Thus, in keeping with the first two phases of the MRC framework, the present study aims to.

1. Evaluate the recruitment process
2. Evaluate the acceptability of the CAHY intervention
3. Determine intervention suitability and participant contentment with the intervention
4. Evaluate and provide preliminary results on symptom changes following the intervention, including anxiety, depression, and physical activity

## Methods and design

Confident Active Happy Youth (CAHY) was evaluated using an open-label feasibility study. The study took place from 19th of August 2021 to 11th November 2022, including baseline assessments, post-intervention assessment, and a 6-month follow-up which was conducted from mid to late 2022.

### Participants

The inclusion criteria for participants were the following:

- Age 8–17 years.
- Symptoms of anxiety and/or depression are assessed by the referring therapist with standardized anxiety and depression questionnaires.
- Youth displaying reduced daily physical activity (less than 30 min per day and/or does not partake in physical leisure activities, and/or does not participate in physical education in school).
- The youth was motivated to partake in physical activity assessed by self-report.

The exclusion criteria were the following:

- Physical activity was not advised for medical reasons.
- Severe learning disabilities and the youth were unable to understand the intervention instructions (e.g., severe learning disabilities).
- Severe psychiatric disorders, including any eating disorder and psychosis.
- Severe challenging behavior or other needs make group participation challenging.

#### Intervention

The CAHY intervention targets core symptoms of anxiety and depression, aiming to help youth become more confident, happy, and physically active, and reduce symptoms of anxiety and depression. The intervention is based on self-determination theory, a theory of interaction between cognition, affect, and behavior (inhibitory-learning theory), and known effects of physical activity on youth (biological, psychosocial, and behavioral: Kodal et al. 2022).

The intervention is a therapist-led, group-based physical activity (PA) program involving aerobic exercise bi-weekly over a course of 7 weeks with two sessions of 50 min per week, except for the last session, which is 3-h long. The groups include up to eight participants with two therapists leading each group. Groups are divided into a child group (aged 8–13: CAHY 8 − 13) and an adolescent group (aged 14–17: CAHY 14–17) to accommodate developmental differences. Both versions of the program follow the same structure and include the same guiding principles and goals. Each session follows the same structure, except the last session which includes a nature hike and a conclusion of the program. This structure for each session consists of an introduction and warm-up (10 min), main activity (35 min), and a session wrap-up (5 min). The separate sessions have discrete overarching topics and include a variety of activities; physical activity (PA) using a ball, PA in a swimming pool, PA focusing on strength, cardio-respiratory focused activities, movement joy, climbing, and an outdoor activity. The sessions comprise a mix of aerobic (e.g., running, jumping, traversing an obstacle course), resistance (e.g., squats, push-ups), and relaxation exercises (e.g., yoga exercises). Sessions take place in a gym hall; two sessions involve activities in a swimming pool and the final session is outdoors. Details of the intervention are described elsewhere [18].

#### Therapists

Two group leaders (*N* = 2), a mental health nurse, and a youth physiotherapist delivered the intervention. Both therapists received training in the specific components of the intervention and received supervision bi-weekly during the intervention period. Supervision was provided by the study principal investigator (AK). The CAHY therapists had experience with working with child and adolescent mental health over several years (4 and 14 years).

#### Measures

Demographic data, including participant age, sex, school attendance, school physical activity attendance, recreational physical activity, and parent social class was gathered using a questionnaire developed specifically for this study. Parent social class was classified in accordance with the Registrar General Social Class coding scheme [19] and was defined by the highest-ranking parent. Social class was dichotomized into the categories of high/medium and low.

Participant pre-treatment motivation to partake in the intervention was *assessed* using the Nijmegen Motivation List Child (NML-C) [20]. The NML-C consists of 15 items rated on a 3-point scale (0 = not at all true, 2 = true). Higher scores indicate higher treatment motivation.

Participant’s mental health information was acquired from the participant’s medical records in CAMHS by the study Principal Investigator (PI). The length of participant treatment in CAMHS was registered in months from their last referral to CAMHS and until participation in CAHY. Participant Axis I psychiatric diagnoses (ICD-10 codes) were grouped into the following sub-groups: *depressive disorders*, *anxiety disorders*, *ADHD disorders*, *pervasive developmental disorders,* and *other disorders* (different psychiatric disorders with prevalence, including bipolar disorder and schizophrenia).

#### Feasibility outcomes

- The recruitment process was assessed through registration of the number of potential participants referred to the study, the number of youths evaluated as eligible, and the number of youths consenting to participate in the study.
- Acceptability of the intervention was examined by way of assessment of treatment participant *retention* rate (treatment completers), treatment *adherence* (number of CAHY sessions delivered), and participant *attendance* rates (participant’s attendance in sessions and in terms of attendance across session categories and reasons for non-attendance).
- Suitability of the intervention was examined in terms of participant feedback regarding the time of day the intervention was delivered, the total number of sessions, and the location of the intervention/traveling distance to the intervention. Feedback was gathered via a self-report.
- Participant and caregiver contentment with the intervention was assessed using a self-report questionnaire. Contentment outcome assessed six aspects of the intervention using a 6-point Likert scale with responses ranging from “very satisfied” through to “very dissatisfied” including the answer option of “I don’t know” (see Additional file 1). Contentment with the intervention was also assessed with an open-ended question examining whether the intervention lived up to expectations for the caregiver and youth. Finally, intervention relevance was assessed, with the categorical question, of whether the caregiver and youth would recommend the program to youth with similar mental health issues.

#### Secondary outcome measures

*Anxiety symptoms* were assessed using the Spence Child Anxiety Scale, child and parent version (SCAS-C/P; Spence, 1998). The SCAS consists of 38 items rated on a 4-point scale (0 = never, 1 = sometimes, 2 = often, 3 = always), with a maximum score of 114. SCAS-C/P has demonstrated good psychometric qualities [21].

*Depressive symptoms* were assessed using *the* Short Mood and Feelings Questionnaire, child and parent version (SMFQ-C/P) (Angold, Costello, Messer, and Pickles, 1995). The SMFQ consists of 13 items rated on a 3-point Likert scale (0 = not true, 1 = sometimes true, 2 = true) with a maximum score of 26. The SMFQ has demonstrated good psychometric qualities [22].

*Self-reported physical activity* was assessed using a self-report questionnaire developed for this study, which examined school physical activity (school attendance categorized as *yes/no*, school PA attendance rated on a 4-point scale ((0 = never, 1 = sometimes, 2 = often, 3 = always) and recreational physical activity assessed in terms of no change, an increase or decrease post-intervention.

*Accelerometery measures of physical activity* were collected using the wearable activity sensor Actigraph GT3X + monitor (Actigraph, LLC, Pensacola, FL, USA). The sensor captures and records continuous physical activity and information about the sleep/wake cycle. This is assessed to be a valid and reliable method [23]. Pre-defined minimum wear time in order for a day to be counted as valid, was defined as 6 h per day, with a minimum of 3 days of data required for analysis inclusion [24]. Actigraph data was downloaded using Actigraph Actilife software (version 6.13.3) and interpreted using 30-s epochs and the following cut-off points: sedentary (< 100 cpm), moderate to vigorous physical activity (MVPA ≥ 2000 cpm). Non-wear time was defined as 60 min of consecutive zero counts. Participants were also asked to wear the Actigraph during each intervention session to assess the amount of MVPA being delivered in each session.

*Biometric data* in the form of weight and height were assessed using a designated weight scale measuring kilograms and a wall-mounted height scale measuring centimeters. Both measures were rounded up to 0.1 kg and 0.1 cm respectively.

#### Progression criteria and adverse events

The following a priori criteria for progression to a definitive study were stated: (A) no serious adverse events, such as hospitalization, a life-threatening condition, death, or any other harmful adverse events associated with the intervention; (B) a recruitment rate of no less than 75%; and (C) a retention rate of no less than 60% in each group to the end of the intervention. If all three criteria were met, this justified the move to a definitive trial, while if one or more criteria were not met, this would justify a “STOP” entailing an assessment of cause and potential remedy. Procedures were in place to handle any possible harms and adverse events related to participation (Kodal et al. 2022).

#### Sample size

A sample of 19 participants was estimated to be sufficiently large to evaluate our metrics of feasibility that map onto our progression criteria; recruitment rate, retention rate, and intervention-related harm [18]. With regard to the likelihood of participants not consenting to participate, we assessed this to be in the order of 30%, whereas the likelihood of drop-out, based on previous meta-analysis findings [25] and results from a similar study [26] was estimated to be 15%. With a confidence value of 95%, and using the equation for sample size calculation suggested by Viechtbauer et al. [27] this results in a sample size of respectively *N* = 9 and *N* = 19 to assess recruitment and retention rates respectively. The largest calculated sample size determines the required number of participants, thus in our case 19 participants, which is estimated as a large enough sample for the problem to occur [27]. Thus, given our progression criteria, we intended recruitment of at least 14 participants (rate = 75% of 19; 95% CI [52%, 95%]), and retention of at least 11 participants (rate = 60% of 19; 95% CI [33%, 88%]). With regard to intervention related harm, we assessed the likelihood of such an event to be extremely low with a progression criterion set to “0” (i.e., π < 0.01). This would entail a very large *N*, which was logistically and practically not possible. Furthermore, in the event of intervention-related harm, the study has sufficient resources to handle such an incident on an as-needed basis, without disrupting or jeopardizing the study.

#### Statistics and data analysis

Quantitative data was analysed using SPSS version 26.0 for Windows (SPSS Inc., Chicago, IL, USA). Analysis of the three progression criteria was performed as follows: recruitment rate was calculated by dividing the number of participants assessed eligible and consenting/assenting to participate, with the total number of eligible participants. Retention rate was calculated as total number of treatment completers divided by all potential treatment completers (youth giving consent/assent to participate) and adverse events was calculated by dividing the incidence of reported adverse events by the total number of sessions (x/sessions). All progression criteria analyses are expressed as percentages and confidence intervals and compared to the a-priori cut-off points. The feasibility study is underpowered to detect any treatment effects, so outcomes will be interpreted only as feasibility data. To assess symptom changes between pre-, post-treatment, and 10-month follow-up, Intention to treat (ITT) analyses with paired mean differences and 95% confidence intervals are calculated [28].

#### Procedure and participation

Youth were recruited from Child and Adolescent Mental Health Services (CAMHS), Department of Child and Adolescent Psychiatry, Haukeland University Hospital, Norway. Patients were referred from the seven outpatient clinics in the/ CAMHS catchment area. Information about the intervention, inclusion, and exclusion criteria was distributed to all therapists in the outpatient clinics by way of information notices on intranet pages and information meetings. The referring clinicians were also encouraged to contact the CAHY team to discuss any uncertainty regarding eligibility. Based on the CAMHS clinician’s clinical assessment of a youth’s eligibility by way of self-report, a referral was sent to the CAHY team. In this referral, clinicians were obligated to document and confirm eligibility criteria among the youths. The CAHY team and Principal Investigator (PI, first author) assessed eligibility. Eligible youth and their primary caregivers were sent an invitation to attend a pre-treatment assessment interview.

In the pre-treatment assessment interview, informed written consent was obtained from all parents and youth aged 16 or above. Assent was obtained from youth who were 12 years old or older. All participants could withdraw their consent with no consequences for continued participation [18]. In the baseline assessment, questionnaires were filled out including demographic data, SCAS-C/P, SMFQ-C/P, and NML-C. Participants were asked to provide height and weight, and all participants were asked to wear an Actigraph activity monitor for the next seven consecutive days, prior to the start of the intervention. During the intervention, participants were also asked to wear Actigraph monitors in every session, apart from swimming sessions.

Following the conclusion of the intervention, participants were invited to attend a post-treatment assessment interview (*M* = 1 week, SD = 1.2). Participants and parents filled out the SCAS-C/P and the SMFQ-C/P questionnaires. Participants were also asked to provide height and weight and to wear an Actigraph activity monitor for seven consecutive days.

Six months post-treatment participants and their parents were invited to attend a follow-up interview. However, circumstances led to the timeframe being shifted to 10 months. The 10-month interview included the SCAS-C/P and SMFQ-C/P questionnaires and feasibility outcomes assessing intervention suitability and contentment.

Participants received one cinema ticket each, and the chance to win a gift certificate (amount = 100 USD) to compensate for their time participating in the follow-up assessment.

Baseline and post-treatment assessments were conducted by the same therapists who delivered the intervention. The use of an Actigraph activity monitor was administered by a research assistant. The 10-month follow-up was conducted by the Principal Investigator (PI).

#### Ethics

Ethical approval for the study was obtained from the Regional Committee for Medical and Health Research Ethics, region West, Norway (no. 30912 REK Vest), and the study was retrospectively registered at ClnicalTrials.gov (no. NCT05049759: 17.09.2021).

## Results

### Recruitment process

**Fig. 1:**
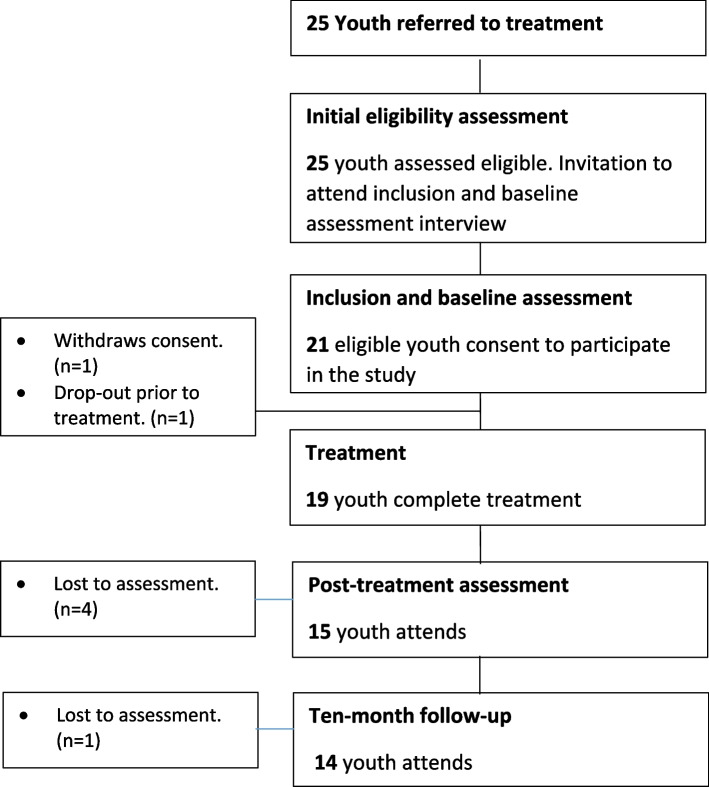
Participant flowchart in the feasibility trial

In total 25 youth were referred to the Confident, Active, and Happy Youth intervention (CAHY) and assessed eligible to attend. Of these 25 youth 21 youth consented to participate in the study. However, one youth later withdrew consent resulting in a total recruitment rate of 80% (20/25: a priori progression cut-off 75%) (Fig. 1).

One youth dropped out of treatment prior to the first session. Since this youth did not receive any treatment, this youth was not included in the study population which thus counted in total 19 or 76% of all eligible participants (meaning 95% of consenting youth). Age distribution among consenting participants lead to a distribution between child and adolescent groups in the order; two CAHY 8–13 groups (*n* = 10) and two CAHY 14–17 groups (*n* = 9).

### Baseline characteristics

Participant characteristics are presented in Table 1. There were differences between participants in the child and adolescent group in clinical measures and diagnoses. Four participants did not attend school at all. Among participants attending school regularly, 47% attend gym class often or always, 20% attend sometimes, and 33% attend rarely or never.

**Table 1:** Baseline characteristics of participants

Pre-treatment characteristics	Participants (*N* = 19)			
	*M*	(SD)	*n*	%
Gender			10	52.6
Age	13.2	2.2		
Age group				
CAHY group age 8–13	11.5	1.2		
CAHY group age 14–17	15.2	1.0		
Family social class				
High/middle			10	52.6
Low			9	47.4
CAMHS diagnosis				
Anxiety disorder			7	36.8
Depressive disorder			5	26.3
ADHD disorder			12	63.2
ASD			2	10.5
Other disorders			7	36.8
Number of comorbid disorders	1.1	1.0		
Treatment time in CAMHS (months)	19.0	12.0		
Psycho-pharmacological medication			10	52.6
Motivation for CAHY (NML-C)	21.1	3.7		
Pre-treatment MVPA (*n* = 16) minutes/day	44.0	21.4		

*ADHD* attention deficit hyperactive disorder, *ASD* autism spectrum disorder, *CAHY* Confident, Active and Happy Youth, *CAMHS* Child and Adolescent Mental Health Clinics, *MVPA* Moderate and vigorous physical activity, *NML-C* Njimegen motivation List- Child. Other mental disorder includes emotional disorder in childhood, learning disabilities, Tourettes syndrome

### Acceptability

Participant *retention* excluding the youth that withdrew consent was 19/20 equalling 95% (a priori progression cut-off: 60% and above the minimum intended retention of *n* = 14). More specifically, the group-wise retention was 89% in CAHY 14–17 (8/9), and 100% in CAHY 8–13 (10/10). Of the 19 treatment completers 15 attended post-treatment assessment equalling 79% and in total 14 youth and their parents participated in the 10-month follow-up (74% of included participants and above the intended retention of minimum 11; follow-up: *M* = 10.1 months post-treatment, SD = 2.9).

Treatment *adherence* (number of CAHY sessions delivered) was 100% (14 sessions *4 groups = 56 sessions). Mean *attendance* across the four groups was 83%, (95% CI [75%,95%]), with no significant difference in attendance between groups or age groups. Excluding airway/Covid symptoms, participant absence was in total 13% of all sessions, meaning overall attendance was 87% (95% CI [79%, 95%]). Differences between participant absences across session themes were not significant, although absence in the final session was app. 50%.

### Suitability

Regarding the time of day, the program was delivered, 63% (10/16) of parents were a little or very satisfied with the time, 19% (3/16) were neither satisfied nor dissatisfied, and 19% (3/16) were a little dissatisfied. The corresponding response among youth was 75% (12/16), 13% (2/16), and 13% (2/16). Parents were also asked if the time of day was an encumbrance to participation, to which 94% (17/18) answered “No”.

In terms of the total number of program sessions, 88% (14/16) of parents were a little or very satisfied with the number, and 13% (2/16) were neither satisfied nor dissatisfied or didn’t know. The corresponding response for youth was respectively 81% (13/16) and 13% (2/16) while 6% (1/16) were very dissatisfied, meaning they experienced the number of sessions as too few.

Parents and youth were also asked if travel distance to the CAHY location a hindrance to participation was. Among parents, 11% (2/18) experienced the travel distance as an encumbrance and four parents commented that transportation to sessions was challenging, although only one family viewed this as a major encumbrance to participation.

Finally, parents and youth were asked if they were content with the pre- and post-intervention assessment. Among parents, 88% (14/16) were little or very satisfied with the assessments, while 12% did not know. Among youth, 75% (12/16) were little or very satisfied with the assessments, whereas 25% did not know.

### Participant and caregiver contentment

Table 2 details youth and caregiver contentment with the program.

**Table 2:** Participant and parent satisfaction with CAHY

Parents (*n* = 16)	Very satisfied	A little satisfied	Neither	A little dissatisfied	Very dissatisfied	Don't know
Satisfaction with the program in general	100					
Satisfaction with the therapists	94					6
Satisfaction with program content	94		6			
Satisfaction with information about CAHY	75	25				
Satisfaction with pre- and post-assessments	81	6				13
Youth (*n* = 16)						
Satisfaction with the program in general	94					6
Satisfaction with the therapists	94					6
Satisfaction with program content	81	19				
Satisfaction with information about CAHY	56	19				25
Satisfaction with pre- and post-assessments	63	12				25

*CAHY* Confident, Active, and Happy Youth. All numbers are presented as percent

Regarding contentment, of the 18 parents who answered, 17 responded that CAHY lived up to expectations. The parent who responded that CAHY did not live up to expectations reported that this was due to a lack of anticipated improvement in mood and anxiety. Of the 16 youth who responded to this question, all responded that the intervention lived up to expectations.

Regarding whether parents and youth would recommend the intervention to other youth with similar mental health issues, among parents (*n* = 15), 14 responded “Yes”, whereas one responded “No”. This response was elaborated with the following statement,” It depends. Participation may influence school negatively (due to less attendance). So, the school should be informed.”

Among youth (*n* = 16), all responded that they would recommend the intervention to a friend.

### Secondary outcomes

#### Anxiety and depressive symptoms

A comparison of means between pre—post- and 10-month follow-up was performed with SCAS child and parent versions, SMFQ child and parent versions below (Table 3).

**Table 3:** Symptom ratings at pre-, post-treatment, and 10-month follow-up. Mean differences and Confidence intervals from pre- to post-treatment and pre- to 10-month follow-up using ITT scores

Measure	Pre-treatment	Post-treatment	10-month FU	Pre-post (*N* = 19)					Pre-10-month FU (*N* = 19)				
							95% CI					95% CI	
	Means	Means	Means	Mean dif	SD	SEM	Lower	Upper	Mean dif	SD	SEM	Lower	Upper
SCAS-C													
Pre-treatment	39.1	40.8	36.2	− 1.79	9.34	2.14	− 6.29	2.71	2.99	12.23	2.81	− 3.00	8.79
SCAS-P													
Pre-treatment	39.0	36.6	32.7	2.32	9.87	2.67	− 2.44	7.07	6.26	12.27	2.81	0.35	12.18
SMFQ-C													
Pre-treatment	9.8	10.3	9.7	− 0.47	2.25	0.52	− 1.56	0.61	0.11	3.86	0.89	− 1.75	1.96
SMFQ-P													
Pre-treatment	9.5	9.9	8.2	− 0.42	4.56	1.05	− 2.62	1.78	1.32	4.8	1.10	− 1.00	3.63

*CAHY* Confident, Active, and Happy Youth, *CI* confidence interval, *FU* follow-up, *SCAS-C/P* Spence Child and Adolescent Anxiety Scale Child/Parent, *SMFQ-C/P* Short mood and feelings questionnaire child/parent

#### Self-reported physical activity

At post-treatment youth were asked yes–no questions on whether they became more confident, active, and happier, following the intervention. Of the 17 respondents at post-treatment, 12 responded they became more confident, 13 became more active, and 11 responded that they became happier. The corresponding parent scores regarding youth were 13, 9, and 13.

Assessed at the 10-month follow-up, nine youths reported they were more at school than pre-intervention, while four reported no change. Among parents, six reported an increase in school attendance, whereas eight reported no change. Regarding school physical activity attendance, 5 youths experienced an increase in attendance following the intervention, two participated less in gym classes and the rest experienced no change (*n* = 14). In terms of recreational physical activity, 10 youths reported becoming more active following TAG (10/14) and nine parents reported their youth had become more active (9/14).

#### Accelerometery

In total, 16 (84%) youth provided baseline accelerometer data, while 7 youths provided accelerometer data at post-treatment (44%). Accelerometer data gathering during sessions and particularly at post-treatment follow-up was subject to data-gathering challenges due to a failure in procedures. However, some data was provided from sessions and post-treatment. Thus, the mean baseline level of moderate to vigorous activity (MVPA) per day was 44 min (SD = 22, *n* = 16) and 31 min (SD = 7.7, *n* = 7) at follow-up. There was no significant difference in daily MVPA between children and youth at baseline or follow-up, or between baseline and follow-up.

Accelerometer data from the CAHY sessions varied according to youth session participation, with an average of 9 measurements per. session (min. 6, max 11). The mean session MVPA was 12 min (min. 9 min, max 20 min, SD = 3.5) with an average of 25% of session time spent in MVPA. Average MVPA varied between sessions ranging between 10 and 15 min (14–30% of session time, SD = 10.1 and SD = 15.0). No significant difference in MVPA was found between sessions or between child and youth participants.

#### Biometric data

Although planned, participant height and weight were not collected, given that most participants (> 75%) declined to provide this information.

#### Adverse events

During the intervention, no adverse events were reported during the intervention equalling 0% (0 of in total 54 sessions delivered). However, the parents of one participant reported that participation in CAHY led to an increase in school refusal. This was reported back to the participant’s attending psychologist, at the local CAMHS, who addressed this issue. Recruitment and retention rates are presented above.

## Discussion

The present study is one of the first studies to test a transdiagnostic physical activity program in a clinical setting. The feasibility of the Confident, Active, Happy Youth (CAHY) program was confirmed with satisfactory recruitment, good ratings of acceptability, suitability, and intervention contentment. In terms of symptom changes, only parent-rated anxiety symptoms demonstrated a marked change (positive confidence interval), whereas youth-rated anxiety, depressive symptoms, and physical activity levels did not change markedly. However, both youth and parents reported improvements in school and school physical activity attendance, and an increase in recreational physical activity. Importantly, no serious adverse events were recorded during or following the intervention.

We set a predetermined recruitment target of no less than 75%, which the study achieved in terms of absolute percentage and with the cut-off well within the estimated confidence interval. Two youths dropped out from the study (9.5%) of whom one withdrew consent. In a meta-analysis examining the effect of physical activity on youth and young adult depression, in a mixed community and clinical population, drop-out rates were in the order of 11%. Thus, our results are on par with this finding, and below the 23% drop-out rate reported in a recent study examining the effect of physical activity on adolescents with anxiety and depression [26].

We set a pre-determined retention rate of a minimum of 60%. Our calculated retention rate was in total 95% (19/20) with the pre-determined cut-off below the estimated confidence interval (85–100%) thus indicating achievement of this progression criteria. All treatment sessions were delivered, and attendance to sessions was high (> 80%), except for the last session (app. 50% attendance). Comparatively, attendance rates in the aforementioned study by Philippot et al. [26] reported a mean attendance rate of 76%. Considering the circumstances that nearly 50% of participants do not attend ordinary gym classes at school, this attendance rate seems promising. The lower attendance rate for the final session may be explained by the length of the session (3 h) and/or that the sessions were outdoors. Given that these sessions took place in autumn/winter, this may have demotivated participants. Similar assumptions with regard to the influence of seasonal weather conditions on participation have been noted in other physical activity interventions with youth [29]. A large-scale study would include a longer recruitment period and therefore recruitment could be mapped to seasons to assess any differences.

The acceptability of the intervention was rated very good in terms of time of day and total number of sessions, although the travel distance to the intervention was challenging for the two families. Some participants had more than a 1.5-h commute to the hospital via rural mountainous roads. Participant and caregiver contentment with intervention content, information, and the pre- and post-assessments were rated as good. Both parents and youth would recommend the program to others with similar mental health issues. This may suggest that the youth experience the intervention as relevant to their challenges and that the intervention achieves creating an environment where the youth dare/are able to participate.

Preliminary changes in mental health symptom scales following the intervention were negligible with one exception. Parent-rated youth anxiety symptoms between pre- and 10-month follow-up demonstrated a marked change, as indicated by the positive confidence interval estimates. While this finding has limited comparable value given the nature of our feasibility study, it is noteworthy that recent meta-analyses have identified some effects of physical activity on both anxiety and depressive symptoms in youth [7–9, 12]. We would have expected to see more change in these measures.

There might be several reasons for the limited changes identified in our study. Participants in the present study consisted of a clinical population, while included populations in the noted meta-analyses are primarily non-clinical community samples [8, 9, 12]. The current study population had on average two clinically diagnosed mental health disorders. Also, the mean treatment time for youth in CAMHS, before referral to CAHY was 19 months, which may be interpreted as a proxy for the severity and complexity of the youth’s mental health issues and disorders. These points underscore one of the main differences commonly highlighted between clinical and non-clinical populations, with the former presenting multiple and more complex symptoms, with corresponding higher functional impairment, including lower levels of physical activity and more barriers towards physical activity [30, 31]. Thus, such increased barriers towards physical activity might have led to less participation in the CAHY intervention, and thus, less exposure to the main intervention elements, including self-determination elements, the exposure, and MVPA (see Kodal et al. 2022 for further details on the intervention). This point is supported by the Actigraph session data that showed a mean participant MVPA per session; of 12 min. This level is both below our intended goal of 30 min [18] and below international recommendations for daily MVPA set at 60 min/day [32]. As such, the participants did not receive an MVPA dosage high enough to lead to the desired changes in their mental health symptoms. Importantly though, the study managed to engage the youth in some MVPA activities, and intervention retention rates were high. Finally, the length and frequency of the intervention may also have been too low to foster symptomatic change. Indeed, the relationship between dose and response (physical activity and health outcome), and potential variations according to type or domain of the activity type is still under researched, not least in clinical populations [8]. A large-scale study including more participants, could help tease out details on this relationship, and potential variations according to the type or domain of the activity and recipient characteristics. However, while no a priori progression criteria were set regarding symptom change and achieved session MVPA levels, the noted findings regarding these variables warrant careful consideration and possible protocol amendment prior to the execution of a large-scale study. To this end, these treatment content variables will be assessed in more detail in a forthcoming qualitative assessment of the feasibility study, which may shed some light on these issues.

Moving on to a large-scale study, considerations should also be made regarding measurement instruments to include. The negligible changes identified on the applied symptom scales may indicate limitations with the current instruments. The anxiety (SCAS-C/P) and depressive (SMFQ-C/P) symptom measurements may not be sufficiently sensitive or specific to capture symptom changes and recovery in participants [33, 34]. In contrast, the self-report questionnaires assessing treatment effects indicated positive changes, including increased confidence (less anxiety), increased school attendance, more school and free-time PA, and better mood. These factors capture functional aspects of the youth’s health, and both complement symptom measures but also represent important outcome measures of youth coping and global functioning. Moving on to a definitive trial, it will be important to include such functional measures in the study, to acquire an extensive assessment of youth’s health and functioning.

Actigraph data was collected during pre- and post-treatment. Three youths declined to use the Actigraph pre-treatment, and most youths (> 60%) used the Actigraph during treatment sessions. Baseline MVPA levels among participants was 43 min. This level is higher than expected yet below international recommendations for daily MVPA in children and adolescents [35]. Compared to national data on mean MVPA levels in youth aged 8–12, the participant MVPA levels are approximately half (43 min versus 90 min) and lower than in an adolescent community sample aged 12–16 (43 min versus 57 min) [36]. Actigraph measurement at post-treatment was impeded by faulty procedures (staff illness) and not by unwillingness among participants. This limits the representativeness of post-treatment data, and the results may be spurious. However, interpreted with this reservation in mind, the mean difference between MVPA levels pre- and post-treatment indicates a decrease in daily MVPA among participants. This was not an intended effect and contrary to our expectations. As previously noted, this may be a result of seasonal weather variations, which has also been identified in previous research demonstrating a significantly higher odds for meeting MVPA requirements in spring as opposed to fall or winter, primarily among younger children and to a lesser degree in adolescents [37]. This points to the need for longitudinal Actigraph follow-up of the participants in future studies, to accommodate for possible seasonal variations.

### Strengths and limitations

The present study has several limitations and strengths. The study is an open-label study. This limits our ability to assess the effects of the intervention itself, versus any effects the youth had from ongoing treatment in CAMHS during and the intervention and the follow-up period. A future study should include a control group. The failure in procedure concerning post-treatment Actigraph assessment hindered analyses of the possible effects of the intervention on youth activity levels. Given this was one of our main outcomes, this is a major limitation of the study. However, the failure in the procedure points to necessary procedural changes to be done before conducting a future study. The use of self-developed questionnaires for youth and parent-reported assessment of functional outcomes limits the ability to compare our results to similar studies.

The study population is heterogeneous, and selection bias is kept to a minimum by employing few exclusion criteria and including youth with multiple and broad mental health issues and disorders. As such, the study population is representative of clinical populations that experience more barriers to *engaging in* and *maintaining* PA compared to community youth populations [30]. Furthermore, the study was delivered in a “real life setting” in a Child and Adolescent Mental Health clinic, and the intervention was delivered by non-specialized therapists. These factors add external validity to the study. Additionally, the study achieved a high participation rate and low drop-out. These rates suggest that a physical activity-based intervention such as CAHY is feasible with a clinical population with comorbid disorders. Another strength of the study is the follow-up period of 10 months. Most studies on the effect of physical activity and youth mental health include post-treatment assessments of effects but very few perform longer-term follow-ups [7, 8, 26]. Long-term follow-up is particularly important with PA interventions, given that continued PA both helps maintain treatment effects and also provides protective effects towards later health issues [5].

## Conclusion

The results of our study indicate that a supplementary trans-diagnostic, physical activity-based intervention is feasible with treatment-seeking youth with anxiety and depression within a clinical setting. Our findings suggest some adjustments are needed to counter the small changes in symptoms, yet all a priori progression criteria were met. Thus, the study provides support, to move on to a definitive trial for which we intend to perform a randomized controlled trial (RCT) of the intervention. A pilot trial to test acceptability to randomization, measurement characteristics and identify appropriate sample size will be performed, prior to the definitive RCT.

### Supplementary Information

**Additional file 1.** Contentment questionnaire.

A**dditional file 2: Table S1.** CONSORT 2010 checklist for feasibility trial.

## Data Availability

The treatment manual for CAHY is available on request (in Norwegian). The dataset supporting the conclusions of this article is not available due to legal regulations.

## Abbreviations

CAMHS: Child and Adolescent Mental Health Care
CAHY: Confident, Active, and Happy Youth
MRC: Medical Council Research Framework
MVPA: Moderate to vigorous physical activity
NML-C: Nijmegen Motivation List Child
PA: Physical activity
PI: Principal Investigator
SCAS-C/P: Spence Child Anxiety Scale, child and parent version
SMFQ-C/P: Short Mood and Feelings Questionnaire, child and parent version
