# Supplementary material for: Feasibility of a physical activity intervention for children and adolescents with anxiety and depression
Source: Pilot Feasibility Stud. 2024 Mar 5;10:49. doi: 10.1186/s40814-024-01466-8 (PMC10913538; doi:10.1186/s40814-024-01466-8)
Supplement: Supplementary file 1 — Additional file 1. Contentment questionnaire. [file 40814_2024_1466_MOESM1_ESM.docx]

**Contentment questionnaire**

Participant and caregiver contentment with the program were assessed with a 6-point Likert scale with responses ranging from “Very satisfied” through to “Very dissatisfied” including the answer option of “I don’t know”. The following questions were administered.

How satisfied are you with CAHY in regards to:

1. CAHY in general?
2. With the information you received about the program?
3. With the therapists?
4. With the programme content?
5. With the time of day for the sessions?
6. With the length of the programme?
7. With the assessment interview pre- and post-treatment
